# Career Aspirations of Medical Students in Germany: the Impact of Gender and Type of Curriculum

**DOI:** 10.1007/s40670-025-02611-5

**Published:** 2025-12-29

**Authors:** Johanna Flora Rother, Alexandra Aster, Tobias Raupach

**Affiliations:** Institute of Medical Education, University Hospital Bonn, Venusberg-Campus 1, 53127 Bonn, Germany

**Keywords:** Medical students, Traditional curriculum, Reformed curriculum, Medical curriculum, Career aspiration

## Abstract

**Background:**

The wide range of possible career choices in the medical field holds both opportunities and challenges. This study investigates which specialties German medical students in their third-year year are considering to take on and investigates differences between students studying in a traditional versus in a reformed curriculum.

**Methods:**

An online multicentre survey was conducted at 23 German medical schools, resulting in 334 participants. A self-constructed questionnaire, which was created by medical experts, was used to examine sociodemographic details and details regarding the curriculum that the participant was enrolled in.

**Results:**

Anesthesiology and intensive care medicine was the most popular specialty among the sample, followed by surgical specialties and internal medicine. Females were more likely to choose obstretics and gynecology while the opposite was the case for internal medicine, which was preferred by male students. Primary care and general medicine was more popular among those students enrolled in a traditional curriculum, compared to those enrolled in reformed curriculum.

**Conclusion:**

The gender differences are line with previous research. The difference between the two types of curricula could be due to the characteristics of the curricula, such as differences in the timing and amount of practical experiences.

## Background

One of the most important and impactful choices of a medical student throughout their education is which specialty they want to pursue. The wide range of career choices and possibilities has been identified as one of the main motivators in German medical students when choosing to study medicine [1], but on the other side, this wide range also highlights the importance of choosing the right specialty.

### Specialty Choices in Different Countries and Cultures

When looking at the specialty choices of medical students, different results have been found in different countries, even though some specialties appear to be generally attractive. To name some examples, a study conducted in Jordan identified surgery as the most attractive specialty [2], similar to results from Rwanda [3]. A systematic review revealed some differences between western and non-western countries, with general practice and anaesthesiology being more attractive in western countries and dermatology and social medicine being more attractive in non-western countries [4]. A recent study at a German university revealed internal medicine, surgery and general practice as the most popular choices across several years of undergraduate education, even though the authors detected a decline in the popularity of surgery closer to graduation [5]. Another study found that up to 50% of students planned to embark on a career in internal medicine and about 25% were interested in surgical specialties [6].

### Influences on Career Choices

Regarding the factors that impact these career choices, several influences and constructs have been discussed. In their systematic review, Levaillant and colleagues found that not only the cultural background influenced the career choices, but also other factors such as gender [4].

Since it is known that gender differences manifest in important motivational factors such as the motives to study medicine [1] and motives are closely aligned with career choices [7], it seems obvious that career choices might differ between genders. A recent worldwide gender-ratio shift in medical students led to a larger percentage of female medical students compared to male students [4, 8]. Therefore, it seems essential to understand these difference and plan education accordingly to avoid possible under- and overstaffing in some professions.

When investigating gender differences, results are mixed. Many previous studies found that male gender was positively associated with a preference for surgery in several studies [9–11]. In line with gender differences regarding motives [1], male participants tend to also put a greater emphasis on career opportunities [11]. On the other hand, a Swedish study found that the only gender difference manifested for a career in gynecology, which was more popular in women than in men [7]. The aforementioned German study found a preference for paediatrics in female students [11]. A different study revealed that 88% of future paediatricians, 82% of future gynecologists and 77% of future general practicioners (GPs) in their sample were female [12]. A study at one single German medical school found that no male students in their sample, no matter which stage of their education they were in, were planning on going into obstetrics and gynecology [5].

The available evidence also indicates that the percentage of students who are not certain about their preferred specialty declines over time [10], suggesting that students gain clarity throughout their undergraduate education, possibly by going through practical experiences and getting a glimpse into multiple specialties. Interestingly, studies also found that female students were often less certain about their specialty choices and made this choice later than their male colleagues [13].

### Curriculum

In Germany, medical schools offer different curricula. The traditional curriculum, which is specified within the German medical licensing regulations [14], is characterized by the division into three sections. Firstly, the preclinical stage (first and second year) which includes seminars, covers basic medical and natural sciences and leads up to the first summative high-stakes exam consisting of a written and an oral-practical examination. The following clinical phase introduces bedside training and covers different medical specialties. This phase ends with a second summative high-stakes exam and is followed by the practical year, which consists of a clinical rotation through three different specialties, at the end of which students take the final summative high-stakes exam to qualify as doctors [14]. In contrast to the traditional curriculum, the possibility of establishing reformed or model curricula has been introduced and applied at several German universities. This gives the universities a chance to reformat their curricula while still providing the students with a degree that is equally recognized as the traditional degree. Model curricula are different from traditional ones in that they usually offer earlier opportunities for patient-contact so that even first- and second-year students can have practical experience through bedside-teaching [15]. Additionally, these reformed or model curricula can differ in their exam formats for the first high-stakes exam, even though the statewide second and third high-stake exams remain the same for all universities.

The differences in career plans between students in different curriculum types have been researched previously, but not extensively. A study conducted by Dettmer and Kuhlmeyer [16] investigated career preferences in last-year German medical students in both curricula. The study was conducted in Berlin, Germany, which offered both curricula. They found that while the top three choices (internal medicine, paediatrics and surgery) did not differ between groups, students in the traditional program preferred internal medicine and paediatrics, while students in the model curriculum favoured internal medicine and gynecology [16]. Interestingly, the authors did not report the percentages of students who aspired to become general practicioners. The well known shortage of general practicioners in Germany [17] highlights the importance of investigating whether the distribution of future general practicioners differs between the students studying in both curricula, since model curricula are becoming the standard at several large universities. Since several other nations, including several European countries [18] and the USA [19] report similar problems regarding a lack of primary care physicians, investigating differences between the different curricula in Germany might be beneficial in order to also translate the results into other countries.

Since the previously mentioned results about curricular influences were produced almost 15 years ago at one German university, this begs the question whether these results still apply and whether they are different when assessing at different universities, compared to one. Consequently, our research questions are as followed:

1. Which career plans do medical students have in their third year?
2. Do the career plans differ between male and female students?
3. Do the career plans differ between those students studying in a traditional German curriculum and those studying in a model curriculum?

## Methods

Along with the previous study by Rother et al. [1] this study is part of the longitudinal research project ‘Transformation of Emotional and Motivational Factors in Medical Students (TEMMS)’, comprising annual data collections at multiple sites across Germany. The data presented here were obtained in the third wave of the survey and analysed independently from the longitudinal data. The study was approved by the local Ethics Committee (application number 2022-342-BO).

### Data Collection

The study was conducted in winter term 2024/25 and data collection took place between October and December 2024. The link to the online questionnaire was sent to over 20 German universities as part of the TEMMS study, which were asked to distribute them among their fifth semester medical students. Additionally, the survey was distributed via an e-mail list where former participants from the first and second survey wave left their contact data in order to be contacted for future survey waves. In the information materials, students were informed that they could use their personal device and that the participation was voluntarily. After clicking on the link or scanning the QR-codes and giving their informed consent, students were directed to the online questionnaire. Completion of the survey took about 10 min. No rewards such as class credits or monetary compensation were awarded to students. At the end of the questionnaire, they were informed that they could either close the tab or click on a link that would take them to a separate questionnaire, where they had the possibility to leave their e-mail address in order to be contacted for future surveys, if they hadn’t done so already.

### Variables and Instruments

The questionnaire consisted of several instruments, which are relevant for the TEMMS longitudinal project. Some instruments are described in Rother et al. [1] and will not be explained here since they are not related to this study.

The demographics were assessed using a self-constructed questionnaire. It included questions about the participants’ age, gender (male, female, diverse), which university, program and curriculum they were enrolled in and several questions regarding their previous education, such as questions about previous patient-contact, (medical) part-time jobs or whether or not they have already worked in the medical field. Finally, the participants were asked whether they already had a specialty or career in mind to choose after graduation. In collaboration with two physicians on the team, twelve categories were determined; ten career options, one category which said “I don’t know yet” and an “Other” category. In an attempt to cover most of the medical field, some categories included more than one specialty. The ten categories (exluding “I don’t know yet” and “Other”) can be found in Table 1.

**Table 1:** Categories regarding the medical career choices

Anesthesiology and Intensive Care Medicine (including Emergency Medicine, Pain Therapy)
Surgical Specialties (e.g. General Surgery, Vascular Surgery, Cardiac Surgery, Neurosurgery, Trauma Surgery, Plastic Surgery)
Dermatology and Venereology (e.g. Aesthetic Dermatology, Allergology)
Obstetrics and Gynecology (including Reproductive Medicine)
Primary Care and General Medicine (including Family Medicine, Preventive Medicine)
Internal Medicine (e.g. Cardiology, Gastroenterology, Pulmonology, Oncology, Nephrology)
Laboratory Medicine, Pathology, and Non-Clinical Disciplines (e.g. Microbiology, Forensic Medicine, Pharmacology, Toxicology)
Pediatrics (Child and Adolescent Medicine)
Psychiatry, Psychotherapy, and Neurology (including Child and Adolescent Psychiatry, Psychosomatics)
Radiology and Nuclear Medicine (e.g. Diagnostic Radiology, Radiation Therapy, Interventional Radiology)

### Data Analysis

IBM SPSS Statistics (Version 29) was used to perform the statistical analysis. After importing the data from Limesurvey and applying the exclusion criteria, the variables were computed and the groups were formed. The participants also self-reported in which type of curriculum they were enrolled in (traditional, reformed, model), which was verified using the universities they reported. From these answers, the participants were divided into either the group studying in a traditional curriculum or in a reformed/model one, which will only be called “model” in the results and discussion section.

As an explorative statistical test, we chose the chi² test of independence, which is able to detect whether two categorical variables are related in a population and therefore can detect significant differences between two groups. In order to perform the test, a variable was calculated to quantify whether the specialty was chosen or not. Each participant could only choose one specialty as their future career plan. Then, the percentages fo each specialty were compared between the groups, using the chi² test of independence.

## Results

### Sample

A total of 508 students started completing the questionnaire, 174 were excluded because they were either not in their fifth semester, did not complete the questionnaire or reported that they were enrolled in another program than human medicine, such as dentistry. This resulted in 334 participants who filled out the questionnaire completely. The mean age in the sample was 22.5 years (SD = 3.0). 238 (71.3%) participants were female, 92 (27.5%) were male, 2 diverse and 2 did not indicate their gender. Due to the small sample sizes in the diverse group, only male and female students were included in the analysis regarding the gender differences. This gender-ratio is in line with the general gender-distribution in German medical students with almost two thirds being female [8]. The final sample consisted of students from 23 different universities, with sample sized ranging from 2 to 40. Out of the 334 participants, 223 (66.8%) were enrolled in a traditional curriculum and 111 (33.2%) were enrolled in a model curriculum.

### Specialty Preferences

**Fig. 1:**
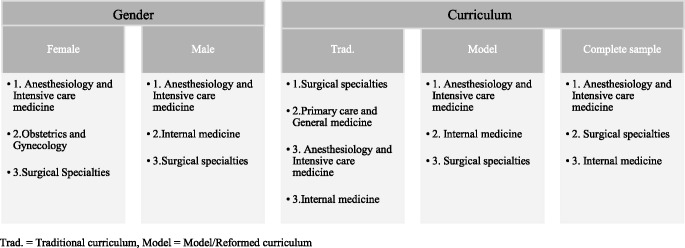
Rankings of the specialties in the relevant group

When looking at the complete sample, over one fifth of the participants reported that they did not yet know which specialty to choose after graduation, while 15% of participants were planning on working in anesthesiology and intensive care medicine, making it the most popular choice. This was followed by any surgical specialty (13.2%) and internal medicine (11.4%). Dermatology and venereology (0.3%) was the least popular choice. An overview of the rankings is given in Fig. 1.

The specialty choices in men and women were somewhat similar, with anesthesiology and intensive care medicine being the most popular specialty for both genders. Interestingly, more women did not have a clear plan about their future career (24.8%) compared to the men in the sample (15.2%), even though this difference did not reach statistical significance. While the second most popular choice among male students was internal medicine (19.6%), it was significantly less popular among female participants (8.4%; χ² (3) = 8.72, *p* = 0.033). A second significant difference was found with regard to obstetrics and gynecology (χ² (3) = 9.31, *p* = 0.025), which was more popular among female students (11.3%), compared to male students (3.3%). Surgical specialties were the third most popular for both groups.

Lastly, we compared the specialty choices between the students studying in the traditional versus model curriculum. Surgical specialties were popular among both groups, being most common in those enrolled in a traditional curriculum (13.9%) and only coming third in those enrolled in a model curriculum (11.7%). The same applies for anestesiology and intensive care medicine, which was the most popular in the participants studying in a model curriculum (19.8%) and second in those studying in a traditional curriculum (12.6%). Lastly, internal medicine reached the third place in those enrolled in a traditional curriculum (10.8%) and the second in those enrolled in a model curriculum (12.6%). A significant difference in distribution between the groups was found for primary care and general medicine (χ² (1) = 8.55, *p* = 0.003). While this specialty came second for the students in the traditional curriculum (12.6%), only 2.7% of those enrolled in the model curriculum were planning to choose this after graduation. An overview of the specialty preferences for the complete sample and the subgroups can be found in Table 2.

**Table 2:** Percentages of the students in the relevant group who chose the indicated specialty

Career choice	Gender (*N* = 334)		Curriculum (*N* = 334)		Complete sample (*N* = 334)
	Female *N* = 238	Male *N* = 92	Trad. *N* = 223	Model *N* = 111	
Anesthesiology and Intensive Care Medicine	12.6%	20.7%	12.6%	19.8%	15%
Surgical Specialties	10.9%	17.4%	13.9%	11.7%	13.2%
Dermatology and Venereology	0%	1.1%	0%	0.9%	0.3%
Obstetrics and Gynecology	11.3%*	3.3%*	9.0%	9.9%	9.3%
Primary Care and General Medicine	10.5%	6.5%	12.6%*	2.7%*	9.3%
Internal Medicine	8.4%*	19.6%*	10.8%	12.6%	11.4%
No Preference	24.8%	15.2%	21.5%	22.5%	21.9%
Laboratory Medicine, Pathology, and Non-Clinical Disciplines	2.1%	1.1%	1.3%	2.7%	1.8%
Pediatrics	10.5%	3.3%	8.5%	8.1%	8.4%
Psychiatry, Psychotherapy, and Neurology	5.0%	3.3%	4.5%	4.5%	4.5%
Radiology and Nuclear Medicine	0.8%	4.3%	1.3%	2.7%	1.8%
Other/did not indicate	2.9%	4.3%	4%	1.8%	0.3%

Trad. = Traditional curriculum, Model = Reformed/model curriculum, * *p* < 0.05

## Discussion

We investigated career choices in medical students from all over Germany. While the most popular choices in our sample were “anesthesiology and intensive care medicine”, “surgical specialties” and “internal medicine”, there were some significant differences between male and female students. Female students were more likely to choose obstetrics and gynecology, while male students were more likely to choose internal medicine. Lastly, students who were currently enrolled in a traditional curriculum were more likely to choose primary care and general medicine, compared to those enrolled in a model curriculum.

Almost one fourth of the sample was still undecided about which career to choose, with more female than male students being undecided. The higher number of female students who were not sure about which specialty to choose, compared to male students, is in line with previous literature [9] and previous research proposed the idea that this might be because some women are also considering motherhood as a possible option [13] while other studies mentioned that family planning nowadays is not only a female issue, compared to previous generations [10]. The preference for gynecology in female students, compared to male students is also in line with previous research and has been explained extensively [5, 7, 12]. The preference of male students for internal medicine is also in line with previous studies [5, 13].

When interpreting the specialty choices in general, it is important to consider the statistics on the actual distribution of physicians among the various specialties in Germany. The popularity of surgery and internal medicine is in line with the official statistics from the German Medical Association [20]. The proportion of participants indicating a specialization in anesthesiology in our study was over twice as high compared to the percentage of actual a anesthesiologists. This raises the question whether this current generation is more drawn towards anesthesiology or whether they change their mind throughout their education.

In 2023, around 10.5% of the working doctors in Germany were general practicioners [20], which is in line with the percentage of participants in our overall sample who were planning on choosing this option. Nonetheless, the significantly lower percentage of those in the model curriculum is alarming, especially given that the structure of medical studies in Germany is currently undergoing significant transformation and traditional curricula are not the norm anymore. Therefore, our study raises the question why students in a model curriculum are less likely to choose general practice and which aspects of the curriculum are responsible for this, especially in light of the shortage of general practicioners in Germany, which might escalate even further in the near future since every third general practicioner is 60 years or older [21].

Our results might have been due to various reasons. Firstly, most students are not able to choose between different universities and might only have one offer for admission. Therefore, this result might simply be accidental. On the other hand, it could be possible that those students who were already planning on choosing general practice apply to medical schools with a traditional curriculum since they are more clearly structured. This might be possibly related to the “down to earth” impression of general practice. Another reason could be that in a traditional curriculum, students mostly gain their practical experience during the clinical year. Consequently, since this questionnaire was conducted with students who had not even completed half of their undergraduate training, it might be possible that the higher popularity of general practice is due to the lack of insights into other specialties. In line with this, a reason for the low popularity of general practise among students in the model program could be that they already caught a glimpse into the work of a general practicioner due to their early practical experience and they already decided against this specialty. This theory is supported by the fact that model curricula offer practical experience earlier on and therefore generates more “competition” between specialties, simply because the students are more informed about the other specialties, even though one could argue that this would manifest in a more equal distribution of the other specialties in this group, which is not necessarily true for our sample.

### Strengths and Limitations

Even though some mechanisms have to be investigated further, the novelty of the comparison between the different curricula is a strength of the study. Another strength is that it was administered with a large germanwide sample and therefore does not only report on students stemming from one university or region, making the results more generalizable. The timing of the study is also a strength, since previous studies often either included students which were at the beginning of their undergraduate education or even before the start of their career, or they focussed on more advanced students such as residents.

This study has some limitations. Firstly, due to the larger choice of specialties and the large percentages of students who were still undecided, the groups used for the comparison tests were relatively small. Future studies might therefore investigate an even larger sample. Also, future studies could further investigate the transformation over time and investigate how many students truly choose the career they plan on choosing during their education. Additionally, it has to be noted that our results only stem from German universities. Future reseach in other countries is needed in order to further understand the effect of curricular differences on career choice. Consequently, our research highlights the need for a clarification study in order to further understand these results in order to avoid possible under- and overstaffing in some professions.

### Practical Implications and Conclusions

Further investigation is needed, whether this lack of popularity of general pratice in model curricula, which are becoming the standard at several large universities, truly results in less general practicioners graduating from these universities. Consequently, it should be made a priority at these medical schools to investigate which aspects of their curriculum might elicit a less favourable image of the general practice. Future studies should therefore focus on investigating specific parts of the curriculum, such as practical experiences in order to find out what could produce these results, especially since the German curriculum differs from other countries. Therefore, the results might also be beneficial to medical educators in other countries in order to gain a better understanding of how curricular differences can influence career choices and how these insights can be used to prevent shortages of some professions. Additionally, interventions should be planned to highten the popularity regarding the specialty choices that were less popular in general, also in order to avoid possible understaffing and, on the other hand, overstaffing in the more popular specialties. There could also be specialized interventions, for example to further introduce specialty choices such as internal medicine to female students in order to avoid unequal gender-ratios in certain specialties. This is especially important in light of the gender-ratio shift.

## Data Availability

The datasets used and/or analysed during the current study are available from the corresponding author upon reasonable request.
